# The emergence of bacterial prions

**DOI:** 10.1371/journal.ppat.1012253

**Published:** 2024-06-13

**Authors:** Rafael Giraldo

**Affiliations:** Department of Microbial Biotechnology, National Center for Biotechnology (CNB-CSIC), Madrid, Spain; National Institutes of Health, UNITED STATES

## Introduction

Prions are proteins that can adopt at least 2 distinct conformations (structural states), being one of them soluble, either natively folded or intrinsically disordered, and the other an aggregated fibrillar structure, termed amyloid, made of stacked ß-strands contributed by the individual protein molecules [1]. Amyloids propagate their conformation to soluble protein molecules of their same kind by contact, capturing them as new building blocks of their elongating fibers. Conformational variants (i.e., alternative 3D folds for the same protein sequence) of an amyloid assembly can be propagated as distinct prion strains vertically with cell division, from mother to daughter cells, for many generations constituting a case for cytoplasmatic, non-mendelian epigenetic inheritance [1]. After the pioneering discovery of the mammalian prion protein PrP [2], the whole field was boosted by findings in the yeast *Saccharomyces cerevisiae*, in which several transcriptional and translational factors (such as Sup35 and Ure2) were shown to aggregate as amyloids, resulting in loss-of-function phenotypes ([*PSI*^+^] and [*URE3*], respectively) [3,4]. Alternative strains of yeast prions were horizontally transmissible between cells while mating through cytoduction (i.e., cytoplasmatic mixing) [5]. In yeast, depending on the protein, its amyloid state either elicits a functionally adaptive phenotypic change or becomes cytotoxic and deleterious [6,7]. Horizontal transmission as an infectious agent has been recently described also for amyloid proteins involved in human neurodegenerative diseases such α-synuclein, Tau, the ß-amyloid peptides, and TDP-43, self-propagating as prion-like proteins (or prionoids) between cells within different areas of the central nervous system [8].

**Fig 1:**
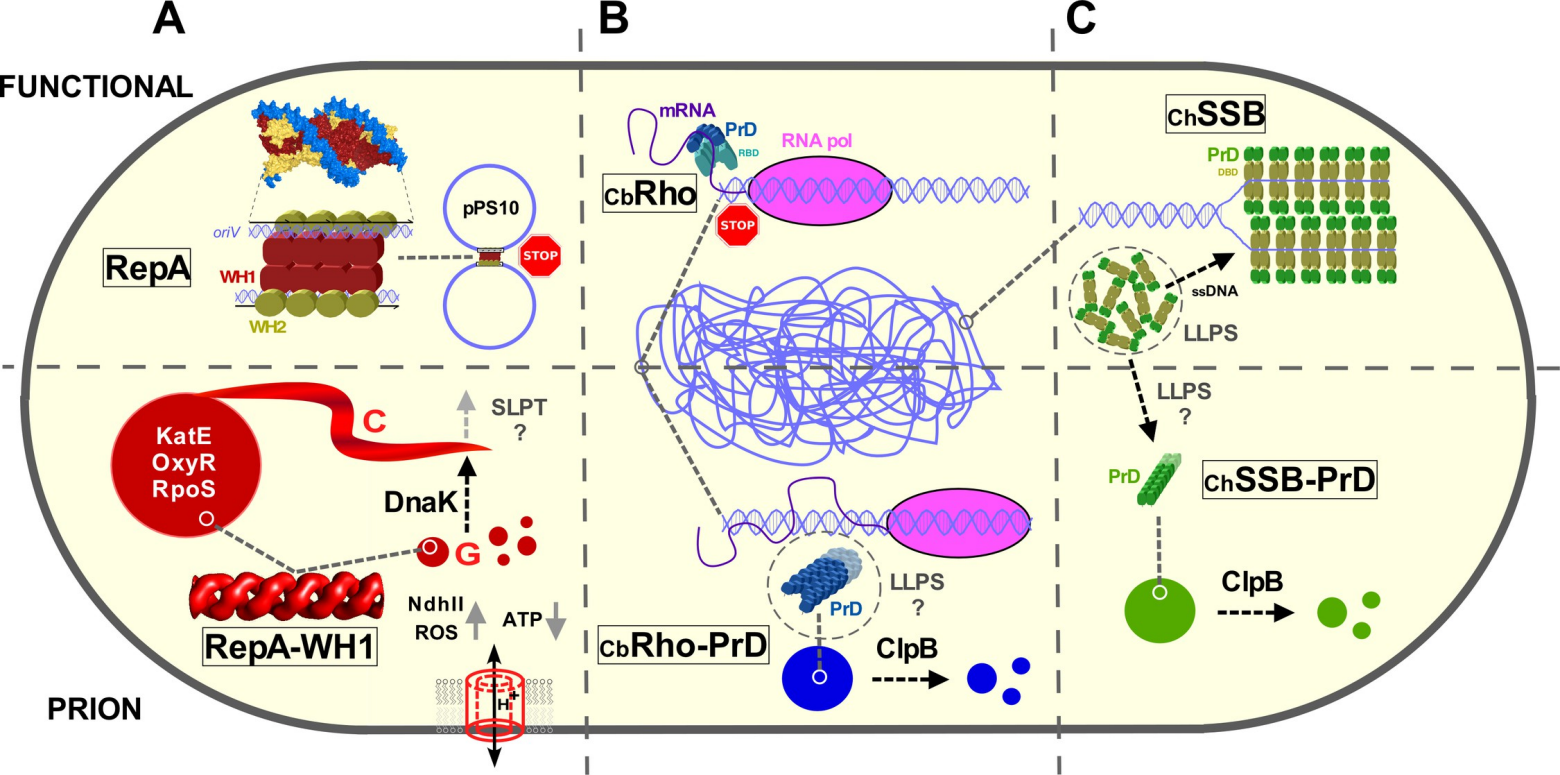
A sketch highlighting the biology of the 3 experimentally characterized bacterial prion proteins, depicted inside a generic bacterium: (**A**) *Pseudomonas* RepA, (**B**) *Clostridium* Rho, (**C**) *Campylobacter* SSB. The functional roles of the native proteins are outlined in the upper half of the panel, while the lower half displays the features associated to their aggregated prion domains (WH1 or PrD; red, blue, and green, respectively). Nucleic acids (ds/ssDNA or mRNA) are their functional targets, while chaperone (DnaK or ClpB)-modulated phase transitions (SLPT) or separations (LLPS) are potential common players in prion strain selection and cell-to-cell propagation. For RepA-WH1, the only one among the 3 prions triggering an amyloid proteinopathy, hits relevant to its cytotoxicity are shown.

In bacteria, as exemplified by the *Escherichia coli* biofilm scaffolding protein CsgA, some extracellular secreted proteins rely on a complex, dedicated protein machinery for their regulated assembly as functional amyloids, with defined fibrillar structures and physiological roles [9]. These are distinct to prions, in which the soluble monomers are functional while the amyloid fibrils that they assemble, usually polymorphic, lose that function [8]. Mining in prokaryotic proteomes identified many proteins with potential prion forming domains (PrDs), typically intrinsically disordered (IDPs) and with overall polar residue composition, similar to those in yeast prions [10,11]. However, so far detailed experimental characterization of bacterial prions is restricted to individual domains in just 3 proteins adopting an amyloid state (Fig 1).

### *Pseudomonas* plasmid replication protein RepA

The N-terminal domain (WH1) in the replication protein RepA of the *Pseudomonas savastanoi* plasmid pPS10 assembles amyloid filaments in vitro upon binding to a specific DNA sequence [12]. In the full-length RepA, when bound to DNA repeats located at the origin of replication (*oriV*), WH1 builds a functional amyloid: an oligomeric superhelical nucleoprotein complex that bridges together 2 copies of the plasmid to hinder their untimely replication [13,14] (Fig 1A). In such oligomers, the WH1 domain is engaged with itself through β-sheet contacts both *in cis*, i.e., between RepA molecules on contiguous DNA repeats, and *in trans*, involving RepA bound to *oriV* from distinct plasmids. A second, C-terminal DNA binding domain (WH2) drives sequence selectivity and affinity for *oriV*.

RepA-WH1 is a natively folded dimeric and stable domain that undergoes a structural transformation promoted by binding to DNA, making a single amyloidogenic sequence stretch accessible to assembly [12,15]. Such amyloidogenic sequence, in spite of being of hydrophobic composition unlike polar PrDs in the other 2 prions discussed here (see below), when cloned as tandem repeats, can replace the Gln/Asn-rich amyloidogenic N-terminal sequences in the yeast protein Sup35, thus maintaining its ability to aggregate as the [*PSI*^+^] prion and enabling stop codon read-through (its natural loss-of-function phenotype) [16].

Fused to a fluorescent protein marker, intracellular RepA-WH1 amyloid aggregates [17] were able to propagate as prions in *E*. *coli* lineages proliferating within a microfluidic chamber [18]. From a mother cell with a polar aggregate, RepA-WH1 generated 2 distinct amyloid strains with defined phenotypes [18]: (i) *G strain*, with several globular aggregates highly cytotoxic, leading to cell filamentation, and highly reactive to an amyloidotropic probe; or (ii) *C strain*, with a single “comet-shaped” particle of a fluid, twisted appearance in time-lapse microscopy, generated from a polar aggregate, that split into 2 with cell division, was less detrimental to cell proliferation and showed reduced reactivity to that amyloid-specific probe. These 2 RepA-WH1 prion strains, as well as prion-free cells expressing the soluble protein with no apparent aggregates (termed Ø), were stably propagated along generations, yet they eventually interconverted with distinct frequencies. The G strain was the dominant final state, leading to cell death [18].

Modulating the expression levels of DnaK, the Hsp70 chaperone of *E*. *coli*, selected for the C over the G strains, while inhibition of DnaK with the phenolic compound myricetin resulted in all cells carrying the G strain. A null mutant for the Hsp104 chaperone ClpB, whether complemented by ClpB expression from a plasmid or not, had no apparent effect on RepA-WH1 propagation [18]. Immuno-electron microscopy of thin sections across the bacterial cells revealed that the C strain aggregates were labeled by both anti-WH1 and anti-DnaK antibodies, as did RepA-WH1 oligomers extracted by DnaK upon incubation with RepA-WH1 fibers preassembled in vitro [18]. This observation strongly supports a role for the Hsp70 chaperone in selecting for the aforementioned fluid and less cytotoxic C-conformation of the prion. It remains to be determined whether such Hsp70-mediated strain-switch towards the more fluid/less compact, yet amyloid-related conformation, represents or not a stage in the full reversion of the liquid-to-solid phase transition that characterizes protein amyloidogenesis [19].

RepA-WH1 prions are cytotoxic in *E*. *coli* by assembling pores at the cell membrane [20]. The resulting membrane leakage reduced the transport of molecules and ATP synthesis, while the compensatory expression of NdhII, a dehydrogenase alternative to complex I in the respiratory chain, triggered oxidative stress [21]. Besides, the G-type aggregates were found to trap some of the proteins essential to mount the response to reactive oxygen species [21]. This pathway, which results in cell division arrest and cell death, resembles findings reported for mitochondria under neurodegeneration [22]. RepA-WH1, which lacks any sequence homologue within Metazoa, can be transmitted horizontally between cultured mammalian cells only if the recipient cells express the bacterial protein as a transgene, thus dismissing possible biosafety concerns [23].

### *Clostridium* transcriptional terminator Rho

Another well-characterized bacterial prion is the N-terminal PrD of *Clostridium botulinum* Rho (*Cb*Rho), an RNA helicase that releases mRNA from its DNA template for transcription termination (Fig 1B). *Cb*Rho-PrD was initially identified as a prion domain in silico [10], and its amyloid nature demonstrated in vitro [24], to be then extensively studied in vivo [11]. As indicated above for the RepA-WH1 amyloid stretch [16], *Cb*Rho-PrD was able to substitute functionally for the Sup35-PrD in yeast, enabling both [*psi*^-^]-like and [*PSI*^+^]-like phenotypes [11]. In *E*. *coli*, a chimeric Rho protein consisting of the *Cb*Rho N-terminal domain (including the PrD) and the catalytically active C-terminal domain from *Ec*Rho was able to support viability as the sole source of Rho [11]. Aggregation of the chimera in the prion form led to the loss of its activity in transcriptional termination, thus allowing for the expression of a *lacZ* reporter situated downstream of a Rho-dependent terminator, which resulted in blue colored colonies on agar [11]. Two distinct colony phenotypes were evident: dark and pale blue, which were attributed to the prion and the non-prion forms of the Rho chimera, respectively. By successive passes on agar plates, it was determined that the dark blue prion phenotype was stably propagated, whereas the pale, non-prion one was quickly lost [11]. Finally, it was shown that propagation of *Cb*Rho-PrD was regulated by the ClpB chaperone, whose overexpression, as for many yeast prions but unlike RepA-WH1 (see above), cured the prion phenotype [11].

The biological significance of the PrD in *Cb*Rho is still unclear, but it is plausible that in its original host the prion state would confer a selective advantage under stress conditions, as conversion to the prion form would reshape the transcriptional program of the bacterium due to genome-wide terminator readthrough. Furthermore, its homologue in *Bacteroides thetaiotaomicron* (*Bt*Rho) has been recently involved in modulating bacterial fitness in the gut of mice under carbon limitation. In vitro, *Bt*Rho underwent liquid–liquid phase separation (LLPS), a physical phenomenon relying on multivalent weak interactions between proteins and nucleic acids [19], thus enhancing transcription termination [25].

### *Campylobacter* single-stranded DNA binding protein SSB

The latest addition to the list of characterized bacterial prions is the PrD of the single-stranded DNA-binding protein from the bacterium *Campylobacter hominis* (*Ch*SSB) (Fig 1C). As for yeast [*PSI*^+^] [26], and for the Sup35 PrD in *E*. *coli* [27], the initial formation of the prion, but not its subsequent propagation, depends on the presence of a heterologous yeast prion protein (New1) that functions as a nucleating factor in its prion form ([*PIN*^+^]) [28]. This was a key finding because it demonstrated prion propagation in the absence of continuous formation of the nucleating prion. In a microfluidic setting, 2 types of cell phenotypes were observed: those with multiple small aggregates, exhibiting low segregational stability leading to the stochastic loss of the prion, and those with a large polar and highly stable aggregate [29]. In addition, a single lineage spontaneously exhibited enhanced partition stability while not carrying any mutation, providing evidence that *Ch*SSB-PrD can form alternative prion strains [28,29], as had been observed with other prion proteins [30]. Neither of these strains seems to express a cytotoxic phenotype in terms of reduction in cell growth rate [29]. Unlike what was observed with RepA-WH1, propagation of *Ch*SSB-PrD depends on ClpB [29], as does Sup35-PrD in *E*. *coli* [31].

Any in vivo function of the *Ch*SSB prion remains uncharacterized, although its homologue *Ec*SSB undergoes LLPS, clustering as droplet reservoirs probably to be quickly mobilized under stress conditions that generate a burst of ssDNA [32].

### Perspectives: The rise of bacterial prions and their potential as antimicrobial agents

Among the crowd of bacterial proteins with the prospect of bearing an intrinsically disordered PrD [10,11], just 2 of them, *Cb*Rho and *Ch*SSB, have been characterized so far in vivo. Besides these, as noted above, RepA includes a folded WH1 domain that also behaves as a prion, which further expands beyond IDPs the potential of proteomes to reach the prion state. Computational approaches to mine proteomes in the search of prions not belonging to IDPs is still missing, although a recent application of AlphaFold opens the way to detection of alternative structures in proteins, which might be a leap forward towards that goal [33]. It is relevant to note that the 3 prion proteins discussed here have been so far studied as synthetic fusions to reporter domains or as interspecies chimeras, not within the context of their wild-type proteins. However, the distinct yet complementary experimental approaches assayed with RepA-WH1, *Cb*Rho and *Ch*SSB will be valuable for the new bacterial prions to come.

A correlation can be established between the unfolded/folded nature of the protein domains and the distinct chaperone requirement for the 2 kinds of bacterial prions: (i) those such as *Cb*Rho and *Ch*SSB with PrDs that are intrinsically disordered, which depend on a Hsp104 chaperone (ClpB) capable of generating propagation-competent seeds through amyloid fiber fragmentation [3]; (ii) the stably folded RepA-WH1, which requires structural remodeling by Hsp70 (DnaK) in conjunction with sequence-specific binding to DNA to expose its amyloidogenic sequence stretch, just as Rep proteins require DnaK (together with the co-chaperones DnaJ and GrpE) to become competent in plasmid replication [34].

It is interesting that the 3 bacterial prions here discussed are intracellular proteins that can experience phase separations and/or transitions, steps in the way to the assembly of hydrogels and amyloids, modulated by chaperones [19]. In addition, they bind to nucleic acids (dsDNA for RepA-WH1, ssDNA in the case of *Ch*SSB, and mRNA for *Cb*Rho) to exert their functions, as for many yeast prions in the context of gene regulation under stress or quickly changing environmental conditions [6]. It is therefore not surprising that bacteria have resorted to the same solution as yeast to achieve efficient (epigenetic) control over central processes involving nucleic acids. If many of the bacterial prions likely “flying under the radar” emerge [10], it might be feasible to ascertain whether the triad outlined here (nucleic acid and chaperone cofactors in phase separation/transition) is a general resource for their performance as prions or not.

There has been a surge in the number of amyloid fiber structures solved by cryo-EM to atomic (or near-atomic) resolutions. Particularly relevant to the subject discussed here are the structures of the fibers assembled by distinct strains of the mammalian prion PrP, which have been recently reported greatly expanding our understanding of the link between structural polymorphism and strain variability. These structures have clarified the basis for host barriers and have even backed up some hypotheses about prion replication and pathogenicity [35]. However, the structure of any of the bacterial prions discussed here is still pending. It is anticipated that such structure(s), when available, will provide important clues about the complex biology of bacterial prions, as reflected in this review. In particular, if those structures reveal how bacterial prions are targeted by chaperones, or how nucleic acids interplay with the assembly of the prion state. The role of ligands in strain selection is still a matter of intense discussion for amyloids involved in human disease, which could be elucidated by information derived from the structure(s) of bacterial prions.

In the wave of the long dispute on the beneficial or pathogenic nature of yeast prions [6,7], it is noteworthy that just RepA-WH1, when decoupled from its role in controlling plasmid replication while in the full-length RepA, shows clear cytotoxicity in bacteria [18,21]. RepA-WH1, *Cb*Rho and *Ch*SSB highlight distinct features of bacterial prions, relevant to prion biology, while RepA-WH1 also constitutes a minimal bacterial model of a “generic” amyloid disease. The understanding derived from such a model might be precious after the recent overwhelming evidence on the ability of amyloids from gut microbiota to nucleate the aggregation of mammalian proteins involved in neurodegeneration, accelerating its progression [36]. In another context, control of the cytotoxicity of the RepA-WH1 prion has been achieved through optogenetics, i.e., the fusion to its N-terminus of a blue light plant photoreceptor domain (LOV2) that, upon illumination, triggered in RepA-WH1 the formation of amyloid oligomers that reduced, as “optobiotics,” the viability of *E*. *coli* [37]. It would be interesting to explore if other homologous RepA-WH1 domains present in the plethora of plasmids spread across bacteria can be used as antibacterials. Through the horizontal transfer of their encoding plasmids or borne by bacteriophages, those prions might target antibiotic-resistant “superbacteria” of the threatening ESKAPE group (*Enterococcus faecium*, *Staphylococcus aureus*, *Klebsiella pneumoniae*, *Acinetobacter baumannii*, *Pseudomonas aeruginosa*, and *Enterobacter* spp.) and even bacterial phytopathogens such as *P*. *savastanoi*, *Xhantomonas* spp., or *Xylella fastidiosa*.
